# Experimental Evidence for Limited *in vivo* Virulence of *Mycobacterium africanum*

**DOI:** 10.3389/fmicb.2019.02102

**Published:** 2019-09-10

**Authors:** Baltazar Cá, Kaori L. Fonseca, Jeremy Sousa, Ana Raquel Maceiras, Diana Machado, Lilica Sanca, Paulo Rabna, Pedro N. S. Rodrigues, Miguel Viveiros, Margarida Saraiva

**Affiliations:** ^1^i3S – Instituto de Investigação e Inovação em Saúde, Porto, Portugal; ^2^Instituto de Biologia Molecular e Celular, Universidade do Porto, Porto, Portugal; ^3^Programa de Pós-Graduação Ciência para o Desenvolvimento, Instituto Gulbenkian de Ciência, Oeiras, Portugal; ^4^Instituto de Ciências Biomédicas Abel Salazar, Universidade do Porto, Porto, Portugal; ^5^Instituto Nacional de Saúde Pública/Projeto de Saúde de Bandim, Bissau, Guinea-Bissau; ^6^Global Health and Tropical Medicine, Instituto de Higiene e Medicina Tropical, Universidade Nova de Lisboa, Lisbon, Portugal

**Keywords:** tuberculosis, *Mycobacterium africanum*, immune response, cytokines, pathology

## Abstract

Tuberculosis remains a public health problem and a main cause of death to humans. Both *Mycobacterium tuberculosis* and *Mycobacterium africanum* cause tuberculosis. In contrast to *M. tuberculosis*, which is geographically spread, *M. africanum* is restricted to West Africa. Differences have also been found in the growth rate and type of disease caused by *M. africanum*, globally suggesting an attenuation of this bacteria. In this study, we used the mouse model of infection to follow the dynamics of *M. africanum* infection in terms of bacterial burdens and tissue pathology, as well as the immune response triggered. Our findings support a lower virulence of *M. africanum* as compared to *M. tuberculosis*, including in mice lacking IFN-γ, a major protective cytokine in tuberculosis. Furthermore, the lung immune response triggered by *M. africanum* infection in wild-type animals was characterized by a discrete influx of leukocytes and a modest transcriptional upregulation of inflammatory mediators. Our findings contribute to elucidate the pathogenesis of *M. africanum*, supporting the hypothesis that this is an attenuated member of the tuberculosis-causing bacteria. Understanding the biology of *M. africanum* and how it interacts with the host to establish infection will have implications for our knowledge of TB and for the development of novel and better tools to control this devastating disease.

## Introduction

Tuberculosis remains a global health problem, with approx. 10 million new cases and over 1.3 million deaths in 2017 (WHO, 2018). Both *Mycobacterium tuberculosis* and *Mycobacterium africanum* can cause human TB (Brites and Gagneux, 2015). These TB-causing bacteria are in turn divided into 7 L belonging to the MTBC, with L1, 2, 3, 4, and 7 encompassing *M. tuberculosis sensu stricto*, and L5 and 6 encompassing *M. africanum* (Gagneux, 2018). Relevant diversity at genomic, clinical and immunological level has been well documented for *M. tuberculosis* (Bastos et al., 2017). Recent studies show that diversity within *M. africanum* L5 and L6 also exists (Ates et al., 2018; Otchere et al., 2018), but its functional impact remains unknown. Interestingly, whereas infections with *M. tuberculosis* are globally widespread, those with *M. africanum* are geographically restricted to West Africa (Yeboah-Manu et al., 2017). Indeed, isolation of *M. africanum* from patients with TB in non-African countries has been mostly restricted to migrants from endemic areas in Africa (Isea-Pena et al., 2012).

The mechanisms underlying the restricted ecological niche of *M. africanum* remain elusive. This restriction may result from specific host-pathogen interactions, as supported by the association of *M. africanum* L5 with TB patients of the Ewe ethnicity in Ghana (Asante-Poku et al., 2015), and by the presence of specific mutations in the genes encoding the ESX secretion system in bacteria of L5 and L6, which could represent adaptations to the niche of the West African host (Winglee et al., 2016). Another possibility relies on *M. africanum* being zoonotic with an animal reservoir limited to West Africa. Infection of animals with *M. africanum* have been described (Thorel, 1980; Alfredsen and Saxegaard, 1992; Gudan et al., 2008), and a novel MTBC strain closely related to L6 has been isolated from a wild chimpanzee (Coscolla et al., 2013). However, evidence of person to person transmission of *M. africanum* has also been reported (Winglee et al., 2016), thus suggesting that an animal reservoir is not fully required. Finally, *M. africanum* may be an attenuated member of the MTBC, and consequently outcompeted by *M. tuberculosis* lineages, in most settings. This last hypothesis is supported by several lines of evidence. Epidemiologically, a decline on the incidence of *M. africanum* has been reported over time for some countries (Dosso et al., 1999; Kallenius et al., 1999; Koro Koro et al., 2013), although not for others (Gehre et al., 2013; Yeboah-Manu et al., 2017). At the clinical level, *M. africanum* has been associated with a slower progression to disease than *M. tuberculosis* (de Jong et al., 2008), and reported to be more commonly found in HIV, malnourished and older individuals (de Jong et al., 2010a). The preferential association of *M. africanum* with HIV remains, however debatable (Meyer et al., 2008). Furthermore, several *M. tuberculosis* key virulence mechanisms have been shown to be affected in *M. africanum* (Yeboah-Manu et al., 2017). Such is the case of the DosR regulon (Ofori-Anyinam et al., 2017), ESAT6 (de Jong et al., 2006), and PhoP/R (Gonzalo-Asensio et al., 2014). Although scarcely explored, data from experimental animal models also point to a slower progression of disease upon infection with *M. africanum* than that observed with *M. tuberculosis* (Bold et al., 2012; Wiens and Ernst, 2016b).

Understanding the course of infection of *M. africanum*, particularly the type of immune responses developed to this member of the MTBC as compared to *M. tuberculosis sensu stricto*, may shed light into novel strategies to tackle TB. In this study, we followed the progression of experimental (mouse) aerosol infection by a clinical isolate of *M. africanum*. As compared to *M. tuberculosis* infections, the clinical isolate of *M. africanum* under study was associated with lower bacterial burdens, signs of disease and tissue pathology, even in hosts lacking IFN-γ, a critical protective molecule in TB. Moreover, the infection was accompanied by a modest cellular and molecular immune response in the lungs of infected mice. Our findings contribute to elucidate the pathogenic potential of *M. africanum*, supporting the hypothesis that *M. africanum* is a less virulent member of the TB-causing bacteria.

## Materials and Methods

### Ethics Statement

Animal experiments were performed in strict accordance with the recommendations of the EU Directive 2010/63/EU and approved by the Portuguese National Authority for Animal Health (DGAV-Ref. 0421/000/000/2016). Mice were kept with food and water *ad libitum* and humanely euthanized by CO_2_ asphyxiation. Every effort was made to minimize suffering.

The human study that originated the blood samples for PBMC isolation was reviewed and approved by the Portuguese Comissão de Ética para a Saúde da ARS Norte (project T792). Written informed consent was obtained for collection of biological material and data from all study participants were anonymized.

### Animals

Eight to twelve-week-old male and female C57BL/6 WT or IFN-γ deficient (^–/–^) mice were bred and housed at i3S animal facility. For infections, animals were housed under contention conditions in the Animal Biosafety Level 3 facility at i3S.

### Bacteria Isolation and Characterization

*Mycobacterium africanum* G67 (Maf_G67) was isolated in Guinea-Bissau at the Instituto Nacional de Saúde Pública (INASA) from a TB patient diagnosed and followed at the Raoul Follereau Hospital, Bissau, Guinea-Bissau, in 2012 (Rabna et al., 2015). It was grown on MGIT tubes for the BACTEC MGIT 960 (Becton-Dickinson, Sparks, MD, United States) according to the manufacturer’s instructions. The Genotype MTBC assay (Hain Lifescience, Nehren, Germany) was used for the differentiation within the MTBC as per manufacturer’s instructions. First-line drug susceptibility testing was performed using the proportion method with the MGIT 960 system, according to the manufacturer’s instructions. Spoligotyping and region of difference (RD) analysis were performed as previously described (Kamerbeek et al., 1997; Rao et al., 2005). Maf_G67 was grown in 7H9 liquid media, supplemented with 10% oleic acid, albumin, dextrose and catalase (OADC) for 7–10 days, with 0.05% Tween 80 for expansion. When in mid-log phase, bacterial stocks were frozen at −80°C in 1 ml aliquots in the presence of 0.2% glycerol. Six aliquots were then thawed and used as control for bacterial load determination by CFU enumeration. The number of viable bacteria obtained after 2 weeks of freezing was 1.7 × 10^8^ CFU/mL, and after 28 weeks of freezing was 1.64 × 10^8^ CFU/mL. Bacterial stocks were washed twice with PBS before aerosol infections.

### *In vitro* Cultures and Infections

Mouse bone marrow-derived macrophages were differentiated from bone marrow precursors in the presence of 20% L929 cell-conditioned media (LCCM). Briefly, bone marrow cells were plated at a concentration of 0.5 × 10^6^ cells/mL in Sterilin Petri dishes in 8 mL of DMEM supplemented with 10% FCS, Glutamine, HEPES and sodium pyruvate and 20% LCCM. On day 4, 10 mL of DMEM with all supplements were added and cells were infected on day 7, at a MOI of 2 (Moreira-Teixeira et al., 2016). THP-1 cells were grown following ATCC instructions, differentiated with 100 nM PMA for 24 h and infected after a 48 h rest using a MOI of 1. Human PBMCs were separated using Histopaque 1077 (Sigma-Aldrich, St. Louis, MO, United States) following the protocol for mononuclear cell separation of SepMate-50 tubes (StemCells, Vancouver, BC, Canada). Cells were frozen and used on the infection day. A MOI of 1 was used, which was calculated to match the total number of cells in culture and which did not compromise cell viability during the infection procedure. At the indicated time points, culture supernatants were harvested and filter-sterilized for cytokine determination by immunoassay (ELISA or Multiplex; eBioscience, Vienna, Austria) or intracellular bacterial load enumeration, as performed previously (Moreira-Teixeira et al., 2016).

### Aerosol Infection and Bacterial Burden Determination

Mice were infected with *M. africanum* via aerosol route using an inhalation exposure system (Glas-Col, United States), as described previously (Moreira-Teixeira et al., 2016). The infection dose was obtained by determining the number of viable bacteria in the lungs of 3–5 mice per experiment, 3 days after infection (<200 CFU for low doses; >500 CFU for high doses of infection). For lung bacterial load determination, organs were aseptically excised, individually homogenized, and serial dilutions plated for CFU enumeration (Moreira-Teixeira et al., 2016). CFUs were counted after 21–28 days incubation at 37°C.

### Histology

Whole lungs were perfused *in situ* with PBS. The right upper lobe was excised, fixed in 3.7% phosphate-buffered formalin for 1 week, embedded in paraffin and cut into 3-mm-thick sections, which were stained with H&E, as previously described (Bhatt et al., 2018). Images were acquired with a NanoZoomer 2.0-HT Whole Slide Imager, Digital Pathology Slide Scanner (Hamamatsu, Japan).

### Cell Population Analysis by Flow Cytometry

Lung single cell suspensions were obtained and stained for surface antigens for 30 min at 4°C. Stained cells were washed and then fixed overnight in PBS containing 2% paraformaldehyde. The following antibodies were used: αCD3-PE (clone 145-2C11, eBioscience), αCD4-PB (clone RM4-5, eBioscience), αCD8-FITC (clone 5H10-1, Biolegend, San Diego, United States), αCD19-APC (clone eBio1D3, eBioscience), αLy6G-APC (clone 1A8, Biolegend), αLy6C-PerCPCy5.5 (clone AL-21, BD Pharmingen), αCD11b-PE (clone M1/70, Biolegend), and αCD11c-PB (clone N418, Biolegend). Dead cells were excluded using Zombie Aqua, a viability dye (Biolegend). All samples were analyzed on a CANTO flow cytometer with Diva Software and data analyzed using FlowJo version 10.1.r7 software. The gating strategies used are shown in Supplementary Figure S1.

### RNA Extraction and Analysis

Total RNA from infected lungs was extracted with TRIzol Reagent (Invitrogen, CA, United States), according to the manufacturer’s instructions. cDNA was synthesized using the SuperScript First-Strand Synthesis System for RT-PCR (ThermoScientific, United States). Target gene mRNA expression was quantified by real-time PCR using SYBR Green (Thermo Fisher) and normalized to ubiquitin mRNA levels, as described before (Bhatt et al., 2018).

### Statistics

Data were analyzed using GraphPad Prism software, version 8.1.0. Student’s *t* test was used to determine differences between two different groups and one-way ANOVA for more than two groups. Post-tests were applied to multiple comparisons as referred in figure legends. Data was checked for normality and log normality. Differences were considered significant for *p* ≤ 0.05 and represented as follows: ^∗^*p* ≤ 0.05; ^∗∗^*p* ≤ 0.01; ^∗∗∗^*p* ≤ 0.001, and ^****^p ≤ 0.0001.

## Results

### *Mycobacterium africanum* Establishes a Slow Progressing Infection With Minimal Tissue Pathology in Immunocompetent Mice

The mechanisms underlying the ecological restriction of *M. africanum* remain unknown, but could be explained by a relative lower virulence of *M. africanum* as compared to *M. tuberculosis*. To gain experimental insight into this question, we decided to investigate the progression of *M. africanum* infection in mice. For this, we used a clinical isolate of *M. africanum* belonging to L6 (AFRI_I), SIT181, and isolated from a TB patient in Guinea Bissau (Rabna et al., 2015), a country that shows the highest prevalence of *M. africanum* recorded in the African continent (Groenheit et al., 2011). This clinical isolate was susceptible to all five first-line anti-TB drugs. According to the RD typing, Maf_G67 groups in *M. africanum* subtype II and belongs to the Guinea-Bissau family (absence of RD7, RD8, RD9, and RD10). As expected, the growth of Maf_G67 in axenic medium was slower as compared to *M. tuberculosis* H37Rv (Supplementary Figure S2).

**FIGURE 1:**
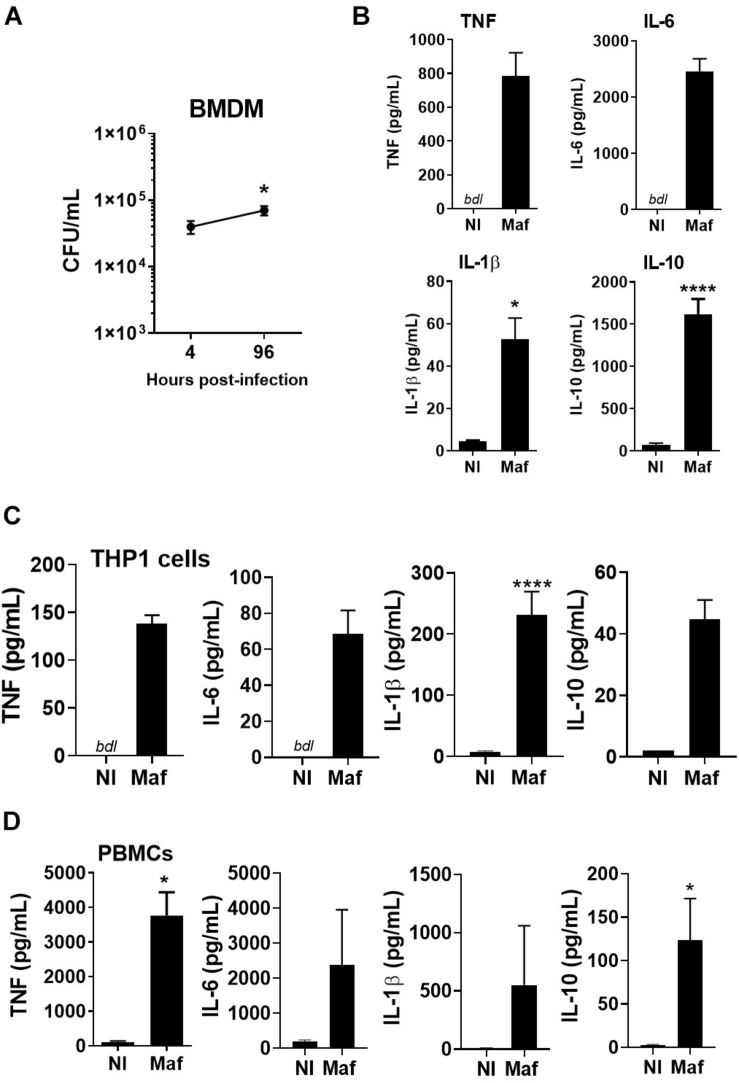
*Mycobacterium africanum* triggers an immune response in mouse and human cells. **(A,B)** BMDM were infected with *M. africanum* (Maf) at a MOI of 2. **(A)** Four and 96 h later the intracellular bacterial load was determined by CFU enumeration. Data is shown as mean ± SEM for three independent experiments, each performed in 4–5 replicate wells. **(B)** Twenty-four hours post-infection, the culture supernatants were harvested and cytokines measured by immunoassay. Data is shown as mean ± SEM for three independent experiments, each performed in triplicate wells. **(C)** THP1 cells were differentiated in the presence of PMA, infected with *M. africanum* at a MOI of 1 and 24 h later cytokine concentration determined by immunoassay. **(D)** Human PBMCs were isolated, infected with *M. africanum* at a MOI of 1 and 24 h later cytokine concentration determined by immunoassay. Data is shown as mean ± SEM for six independent donours. Non-infected (NI) cells are included as controls. Unpaired *t*-test and Mann–Whitney test were used to perform the statistical analysis. ^∗^*p* < 0.05; ^****^p < 0.0001. bdl, below detection level.

We started by infecting macrophages with the *M. africanum* clinical isolate under study. *In vitro* infection of mouse BMDM showed persistence of this isolate over time (Figure 1A) and triggered the secretion of both pro- and anti-inflammatory cytokines (Figure 1B). Cytokine secretion was also observed upon infection of PMA-differentiated THP1 cells, a human monocytic cell line (Figure 1C), or human PBMCs (Figure 1D). The rational underlying the use of different cell types was to test if the clinical isolate used in our study triggered a cytokine response that was not dependent on the cell type. These data show that the *M. africanum* clinical isolate infects, persists in and is recognized by innate immune cells in spite of their different origin and genetic background.

**FIGURE 2:**
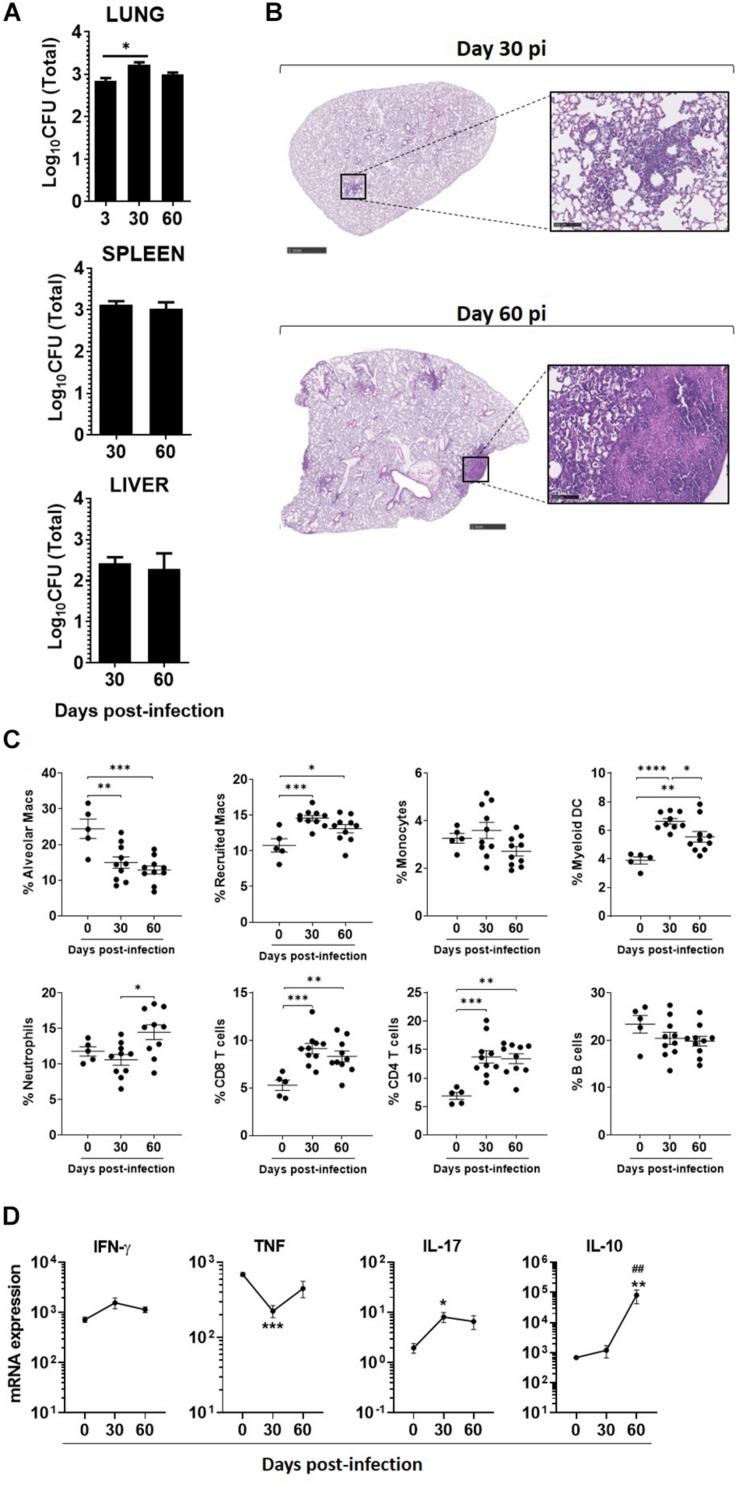
*Mycobacterium africanum* infection is controlled by immunocompetent hosts, with minimal pathology and immune responses. C57BL/6 mice were infected via aerosol with a high dose (>500 CFU) of *M. africanum*. **(A)** At the indicated time points, the lungs, spleens, and livers of infected mice were collected and the bacterial burden determined by CFU enumeration. Lungs were harvested at the indicated time points and **(B)** pathology determined by H&E staining; **(C)** immune cell populations determined by flow cytometry; and **(D)** the expression of the indicated cytokines measured by real-time PCR. Data are shown as mean ± SEM from at least two independent experiments with five animals each. The images in **(B)** are of one animal representative of the experimental group. Scale bar on the left and right images correspond to 1 mm and 100 μm, respectively. One-way ANOVA with Tukey’s post-test was used to perform the statistical analysis. ^∗^*p* < 0.05; ^∗^^*,##^*p* < 0.01; ^∗∗∗^*p* < 0.001; ^****^p < 0.0001. Asterisk (^∗^) related to differences compared to NI mice, and hash (#) related to differences comparing d30 to d60 post-infection.

Next, we infected C57BL/6 WT mice with a high dose of *M. africanum* via the aerosol route. A high dose of infection was performed to probe the host ability to deal with *M. africanum* under more extreme conditions than those associated with low doses of infection. *M. africanum* was able to effectively infect and persist in the mouse lungs, but the bacterial burden did not increase significantly over time (Figure 2A). Moreover, the bacterial burdens detected in the lung were substantially lower than those observed in infections with *M. tuberculosis* reference strains (L4, H37Rv; or L2, HN878) (Table 1). In spite of this poor growth in the lung, *M. africanum* disseminated to the spleen and liver over the 60-day time course (Figure 2A).

**TABLE 1:** Comparison of lung bacterial burdens obtained for infections with a *M. africanum* clinical isolate versus *M. tuberculosis* reference strains HN878 and H37Rv.

	**Low dose**		**High dose**	
	**Log_10_CFU**		**Log_10_CFU**	
	**d30**	**d60**	**d30**	**d60**
*M. africanum* (L6)	2.62 ± 0.022	2.45 ± 0.021	3.012 ± 0.129	2.89 ± 0.068
*Mtb* HN878 (L2)	6.078 ± 0.087	5.723 ± 0.110	7.453 ± 0.138	–
*Mtb* H37Rv (L4)	4.866 ± 0.0898	5.024 ± 0.296	–	5.203 ± 0.314

*C57BL/6 mice were infected via aerosol with a low (<200 CFU) or high dose (>500 CFU) of the indicated bacteria and on day 30 or 60 post-infection the lungs were collected and the bacterial burden determined by CFU enumeration. Data are shown as mean ± SEM from at least five animals per time point. Mtb, *M. tuberculosis*; L, lineage.*

Tissue sections of *M. africanum*-infected lungs were examined to assess pulmonary pathology. The extent of inflammatory infiltrates and pathology observed in *M. africanum*-infected animals was minimal (Figure 2B). In the case of *M. tuberculosis* infections, multiple inflammatory foci with infiltration of immune cells and perivascular lymphocyte recruitment was seen (Supplementary Figure S3). These histopathological features, which are characteristic of aerosol infections with high doses of *M. tuberculosis* and accompanied by extensive tissue damage, were mostly inexistent in the case of *M. africanum* infection. On day 30 and 60 post-*M. africanum* infection, the lung tissue was mostly preserved, except in limited areas where a modest immune infiltration was noted (Figure 2B).

### A Discrete Immune Response Develops in the Lung Upon *M. africanum* Infection

In parallel, we investigated the recruitment of myeloid and lymphoid immune cell populations to the lung of infected mice. Overall the recruitment of immune cells was observed over time (Figure 2C). Specifically, a significant, but modest, increase was observed in the lungs of infected mice for recruited macrophages, myeloid DCs, neutrophils, CD4, and CD8 T cells (Figure 2C). This observation supports the activation of an immune response upon *M. africanum* infection.

We also measured the transcription of several immune mediators in the lung of *M. africanum*-infected mice. In line with the modest immune cell recruitment observed (Figure 2C), the transcription of the analyzed molecules was also discrete (Figure 2D). Indeed, as compared to non-infected mice, there was no significant upregulation of *ifng* or *tnf* (Figure 2D) and the transcription of *nos2* was undetected (data not shown). A significant upregulation of *il17* transcription was observed (Figure 2D). Interestingly, in contrast to what was observed for these pro-inflammatory mediators, the transcription of the anti-inflammatory cytokine IL-10 was markedly upregulated during infection (Figure 2D).

Altogether, these data are in line with the bacterial burden and histology findings, which highlight infection in the lung with limited immune activation and pathology.

### Lack of IFN-γ Does Not Compromise the Survival of Mice Infected With *M. africanum*

**FIGURE 3:**
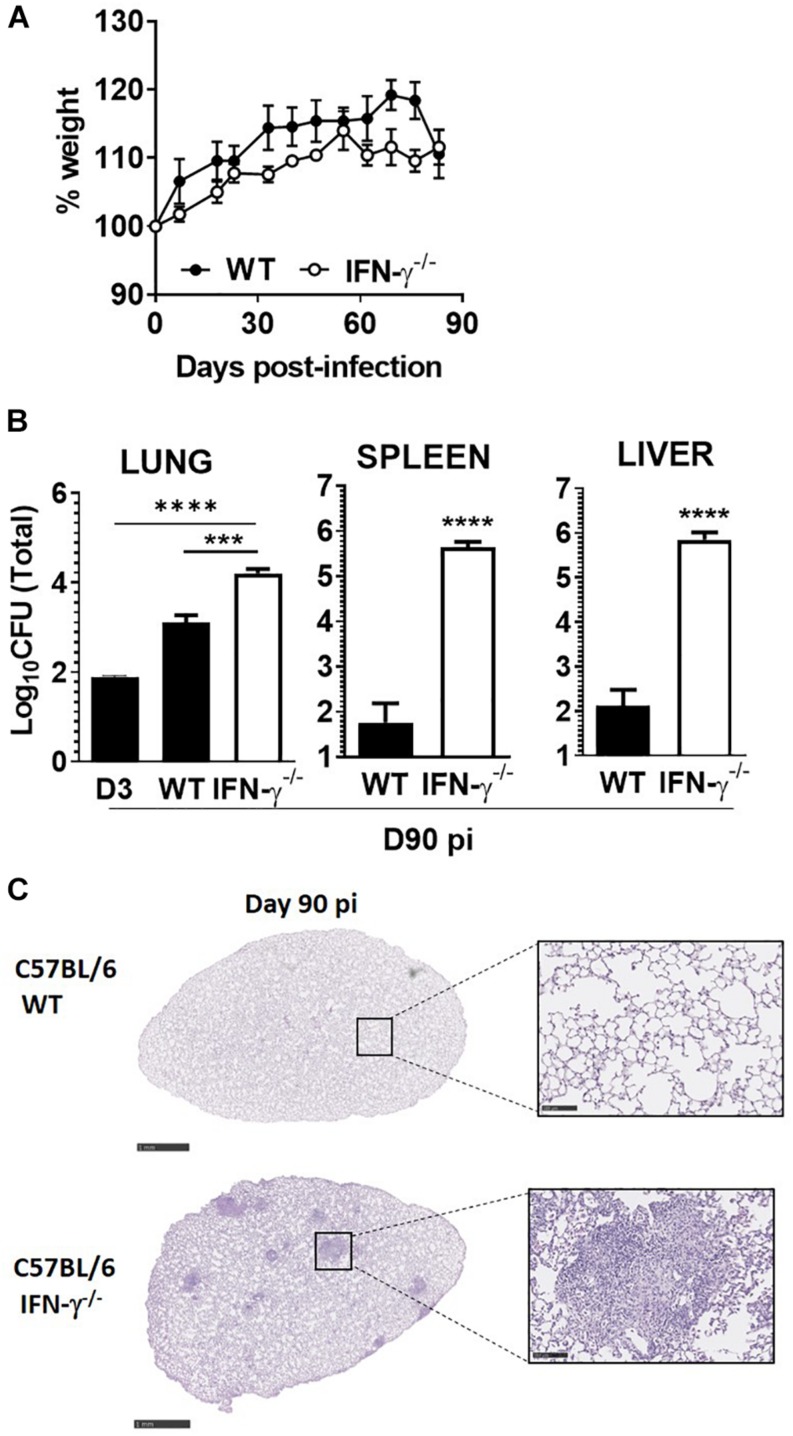
IFN-γ is required for optimal control of *M. africanum*, but its absence does not lead to overt disease. C57BL/6 WT (black circles or bars) and IFN-γ^–/–^ (white circles or bars) mice were infected by aerosol with a low dose (<200 CFU) of *M. africanum*. **(A)** The weight of the animals was monitored weekly up to day 90. On day 90 post-infection, **(B)** lungs, spleens, and livers of infected mice were collected and the bacteria burden determined by CFU enumeration and **(C)** lungs were harvested and pathology determined by H&E staining. Data are shown as mean ± SEM from five animals per group. The images in **(C)** are of one animal representative of the experimental group. Scale bar on the left and right images correspond to 1 mm and 100 μm, respectively. Unpaired *t*-test was used to perform the statistical analysis. ^∗∗∗^*p* < 0.001; ^****^p < 0.0001.

Given our observations in *M. africanum*-infected C57BL/6 immunocompetent mice, we next questioned whether the absence of a major protective molecule would impact the course of infection by this pathogen. For that, we aerosol-infected C57BL/6 WT or IFN-γ^–/–^ mice with the *M. africanum* clinical isolate under study and followed the progression of infection over a period of 90 days. Considering the high susceptibility of IFN-γ^–/–^ mice to *M. tuberculosis* (Cooper et al., 1993; Nandi and Behar, 2011; Moreira-Teixeira et al., 2016), we decided to perform the infection with a low dose of bacteria. Notably, IFN-γ^–/–^ mice infected with *M. africanum* survived for the duration of the experiment, showing no signs of weight loss (Figure 3A). Furthermore, although the lung bacterial burden was significantly increased in IFN-γ^–/–^ infected mice, as compared to WT ones, bacterial growth was still well controlled (Figure 3B) and far from the typical values observed upon aerosol infection of these mice with *M. tuberculosis* (Nandi and Behar, 2011; Moreira-Teixeira et al., 2016). As compared to C57BL/6 WT animals, a higher dissemination of *M. africanum* to the spleen and liver of IFN-γ^–/–^ mice was observed (Figure 3B). Also, in IFN-γ^–/–^ mice, a robust growth of *M. africanum* was seen in these organs as compared to that observed in the lung. At the histologic level, WT lungs showed no detectable signs of tissue pathology (Figure 3C), showing that as compared to infections with high doses of *M. africanum* (Figure 2B), upon a low dose of infection a lesser infiltration of immune cells develops. IFN-γ^–/–^ mice infected with *M. africanum* presented signs of immunopathology in the lung (Figure 3C), with well-localized infiltrates surrounded by areas of healthy tissue.

Collectively, these data suggest that the lack of IFN impacted the ability of mice to deal with *M. africanum* infection, but in contrast to *M. tuberculosis* infections (Cooper et al., 1993; Nandi and Behar, 2011; Moreira-Teixeira et al., 2016), it did not lead to overt disease and premature death, at least during the first 90 days of infection.

## Discussion

Despite the close relationship between *M. africanum* and *M. tuberculosis*, their biology, epidemiology, and potential to cause disease is different, calling for the study of TB disease and associated immune responses to each bacteria. In this study, we resorted to the mouse model to follow the progression of infection by a clinical isolate of *M. africanum* in terms of bacterial burden, tissue pathology and immune response triggered. Our findings suggest a slow progressing of infection, with mild lung pathology, even in typically highly susceptible hosts. The *M. africanum* clinical isolate under study infected, persisted and induced a cytokine response by mouse or human cells in *in vitro* cultures, thus showing its ability to establish infection and to actively interact with host immune cells. The persistence of *M. africanum* in resting macrophages recapitulates previous observations (Homolka et al., 2010).

Our *in vivo* data are compatible with a slower course of infection of at least some isolates of *M. africanum* in the mouse model, which is in line with what has been reported in humans (de Jong et al., 2008) and also with two previously studies using the mouse model (Bold et al., 2012; Wiens and Ernst, 2016b). In one of these former studies (Bold et al., 2012), the reduction of bacterial burden seen in the lungs of *M. africanum-*infected animals, as compared to *M. tuberculosis*, was not as pronounced as we report here. In our study and those previously published (Bold et al., 2012; Wiens and Ernst, 2016b), the *M. africanum* isolates used belonged to L6. However, whereas in our study the clinical isolate has been obtained from a TB patient in Bissau, the previous studies used a bacteria that had been isolated from a TB patient in The Gambia (Bold et al., 2012; Wiens and Ernst, 2016b). It is possible that the variations observed in terms of reported bacterial burdens reflect the recently demonstrated *M. africanum* intrapathogen diversity (Ates et al., 2018; Otchere et al., 2018), and so that a spectrum of virulence exists among *M. africanum* isolates. It will be important to address this possible spectrum of *M. africanum* virulence, as it may explain several apparent contradictory observations. For example, a possible attenuation of *M. africanum* supports the likely outcompetition of this pathogen by *M. tuberculosis*, as suggested by studies reporting a decreased incidence of *M. africanum* over the time (Koro Koro et al., 2013). However, in other geographic locations, the incidence of *M. africanum* appears to be constant (Yeboah-Manu et al., 2017; Asare et al., 2018) and a recent study including 3580 isolates from 12 different countries (Gehre et al., 2016) show that *M. africanum*, particularly L6, remains a significant cause of TB in West Africa.

Our findings reveal for the first time that infection with a *M. africanum* clinical isolate causes a mild lung pathology upon infection with a high dose of bacteria and over a 60 days period. This is in sharp contrast to what we and others have observed for infections with *M. tuberculosis* isolates and may reflect the slow progression to TB disease described in humans infected with *M. africanum* (de Jong et al., 2008), and also in some animals (de Jong et al., 2010b). In turn, the slow progression of *M. africanum* infection may be related to the delayed growth described for these bacteria using *in vitro* methods (Gehre et al., 2013) or to a certain impairment to grow in the lung, reflective of a microaerobic preference developed by *M. africanum* when compared to *M. tuberculosis* (Ofori-Anyinam et al., 2017). Our observation is important as it suggests that, as compared with *M. tuberculosis* infection, in humans infected with *M. africanum*, lung damage and eventually cavitation may take a longer time to develop. However, it is important to refer that at presentation the disease caused by *M. africanum* is at least as severe as that caused by *M. tuberculosis* (de Jong et al., 2007; Tientcheu et al., 2016). Still, a slower progression to disease may eventually compromise the transmission of *M. africanum* in the long term. Indeed, a recent study showed that in Ghana the transmission of *M. africanum* seems to be lower than that of *M. tuberculosis* (Asare et al., 2018), whereas in Mali person to person recent transmission has been reported (Winglee et al., 2016) and in The Gambia similar rates of transmission between *M. tuberculosis* and *M. africanum* have been described (de Jong et al., 2008). These variations may be related to the host and pathogen intrinsic diversity.

Furthermore, we show the dissemination of this *M. africanum* isolate to the spleen and liver of infected animals, where it appears to grow somehow better than in the lung. Several reasons may explain the dissemination of a bacteria that is not growing well in the lung. On one hand, it is possible that the lack of a robust T cell response may allow the escape of more bacteria from the lung to other organs. In HIV-TB co-infected patients, where CD4 T cell responses are lacking, a higher dissemination of *M. tuberculosis* occurs (Aaron et al., 2004). On the other hand, the more competent growth of *M. africanum* in the spleen and liver, as compared to the lung, may be a consequence of the downregulation of DosR and preference for microaerobic growth described for this bacteria (Ofori-Anyinam et al., 2017). In conclusion, our observations offer the hypothesis that TB caused by *M. africanum* may be associated with higher rates of extra-pulmonary or disseminated disease, as suggested in a recent study (Sharma et al., 2016). They also indicate a reduced degree of virulence of *M. africanum*, which may be related to dissemination, rather than lung damage, during early stages of infection.

Moreover, the control of this *M. africanum* isolate in the mouse lung is reflected in the low recruitment of immune cells and induction of cytokine transcription. Indeed, the transcription of key cytokines as IFN-γ, IL-17, and TNF, as well as NOS2, in the lungs of infected over non-infected mice was barely detected. Noteworthy, however, was the robust transcription of IL-10, which production was also seen in *in vitro* infected mouse or human macrophages, and human PBMCs. It will be interesting to, in the future, further dissect the mechanisms regulating IL-10 expression upon infections with different isolates of *M. africanum*, as well as to elucidate the possible contribution of IL-10 for *M. africanum* pathogenesis, both in the context of immunocompetent or immunocompromised mice. Furthermore, it will be also interesting to establish the dynamic of immune cell recruitment during the initial phases of infection, when the innate immune responses are likely predominant. Another outstanding question relates to the mechanisms underlying the lower virulence associated with *M. africanum*, which remain undisclosed. Of note, the interaction of *M. africanum* with mouse macrophages has been shown to differ from that of *M. tuberculosis*, in what regards the transcriptional reprograming of the bacteria (Homolka et al., 2010), as well as the triggering of mitochondrial ROS and ultimately of type I IFN (Wiens and Ernst, 2016a). How these observations articulate and whether they may contribute to the lower virulence of *M. africanum* is unknown.

The overwhelming protective nature of IFN-γ in TB has been extensively described in mice and humans. IFN-γ^–/–^ mice infected with *M. tuberculosis* typically succumb to infection during the first 40 days (Cooper et al., 1993; Nandi and Behar, 2011; Moreira-Teixeira et al., 2016), whereas humans with mutations in the IFN-γ producing/responding axis present Mendelian susceptibility to mycobacterial disease (Abel et al., 2018). Thus, the finding presented here for the first time that mice lacking IFN-γ coped well with *M. africanum* aerosol infection was most surprising. Still, it is important to refer that although IFN-γ^–/–^ mice survived a low dose infection with this isolate of *M. africanum* for at least 90 days, they did present higher lung bacterial burden and evidence for immune pathology, not seen in IFN-γ competent mice. This indicates that similar protective mechanisms operate among human-adapted TB causing bacteria, albeit their required threshold and dynamics for host protection are different. In the same context, a recent report demonstrated that type I IFN receptor signaling is detrimental during infections with a different isolate of *M. africanum* (Wiens and Ernst, 2016b), as previously reported for *M. tuberculosis* (Berry et al., 2010; Mayer-Barber et al., 2014). Also, for both TB patients and household contacts, *M. africanum* infection has been shown to elicit diminished T cell responses to ESAT6, as compared to those elicited by *M. tuberculosis* (de Jong et al., 2006). A subsequent study identified higher proportion of single TNF and lower proportion of single-IL-2-producing CD4 and CD8 T cells in *M. africanum*-infected patients, as compared with *M. tuberculosis*-infected ones before antibiotherapy, and persistently high proportion of activated T cells in *M. africanum* infected patients after treatment (Tientcheu et al., 2014).

In conclusion, several recent studies, including the one presented here, are bridging the gap relatively to our limited knowledge on *M. africanum* and pointing to an overall lower virulence of these bacteria. The mechanisms underlying this lower virulence are not fully understood, highlighting the need for more research using diverse *M. africanum* isolates, including those belonging to L5. Understanding whether *M. africanum* is indeed less virulent and the underlying causes, may shed important clues on how to ≪attenuate≫ *M. tuberculosis*, either by targeting the bacteria or the host immune response (Zumla et al., 2017). Furthermore, a deep understanding of *M. africanum* infections will inform the design of common or specific vaccines and therapies.

## Data Availability

The datasets generated for this study are available on request to the corresponding author.

## Ethics Statement

The human study that originated the blood samples for peripheral blood mononuclear cells (PBMC) isolation was reviewed and approved by the Portuguese Comissão de Ética para a Saúde da ARS Norte (project T792). The patients/participants provided their written informed consent to participate in this study. Animal experiments were performed in strict accordance with the recommendations of the EU Directive 2010/63/EU and approved by the Portuguese National Authority for Animal Health (DGAV-Ref. 0421/000/000/2016).

## Author Contributions

BC, KF, JS, AM, and DM performed the experiments. BC and KF analyzed the data and wrote the manuscript. LS, PRa, and MV performed the work leading to the isolation of the clinical strain of *M. africanum* used. PRo and MV wrote the manuscript. MS supervised the study, planned the experiments, analyzed the data, and wrote the manuscript.

## Conflict of Interest Statement

The authors declare that the research was conducted in the absence of any commercial or financial relationships that could be construed as a potential conflict of interest.

## Supplementary Material

FIGURE S1 Gating strategy used to delineate the cellular populations present in the lung.

FIGURE S2 *In vitro* growth rates of *M. africanum* G67 compared to *M. tuberculosis* H37Rv. Bacterial suspensions were adjusted to 10^6^ CFU/ml with a sterile saline solution and 0.5 ml transferred into a MGIT tube containing 10% OADC. Growth rates were measured at 37°C with the MGIT 960 and the Epicenter software for 28 days.

FIGURE S3 Infection of C57BL/6 mice with a high dose of *M. tuberculosis*. C57BL/6 mice were infected via aerosol with a high dose (>500 CFU) of *M. tuberculosis* strain HN878. On day 30 post-infection, the lungs were collected and pathology determined by H&E staining. The images are of one animal representative of the experimental group (*n* = 5). Scale bar corresponds to 2.5 mm on the left and 100 μm on the right panel.

## Abbreviations

^–/–^: deficient
BMDM: bone marrow-derived macrophages
CFU: colony forming unit
DC: dendritic cell
ESAT6, early secreted antigenic target: 6 kDa
H&E: hematoxylin and eosin
L: lineage
MOI: multiplicity of infection
MTBC: *Mycobacterium tuberculosis* complex
PBMCs: peripheral blood mononuclear cells
TB: tuberculosis.
