# Supplementary figures and images for: Experimental Evidence for Limited in vivo Virulence of Mycobacterium africanum
Source: Front Microbiol. 2019 Sep 10;10:2102. doi: 10.3389/fmicb.2019.02102 (PMC6746983; doi:10.3389/fmicb.2019.02102)

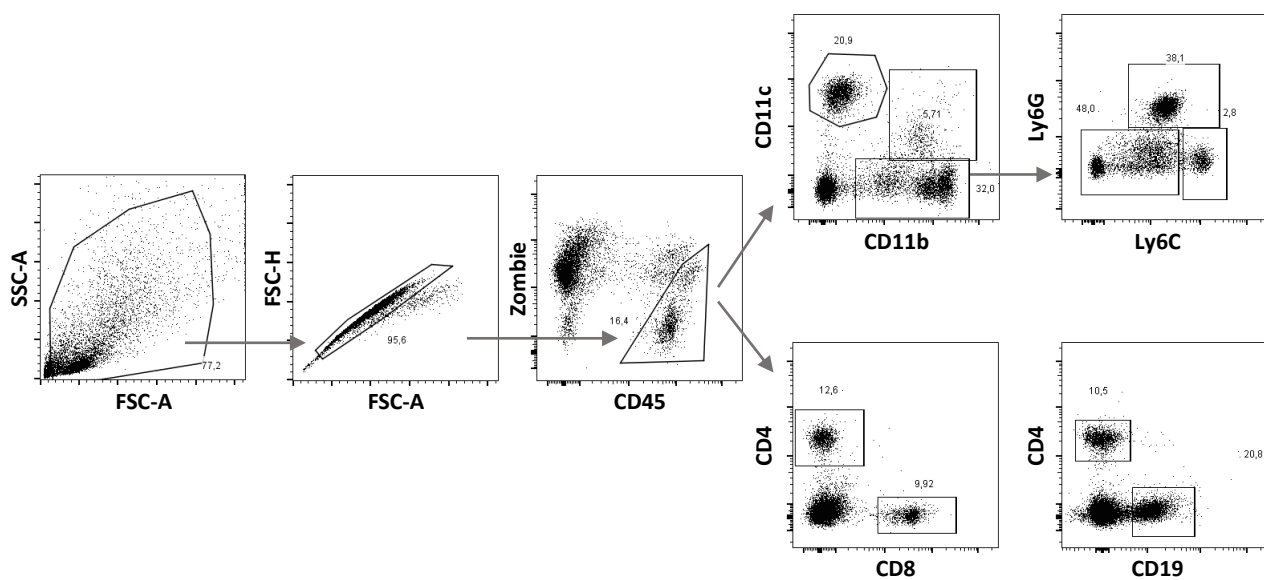

Supp Figure 1

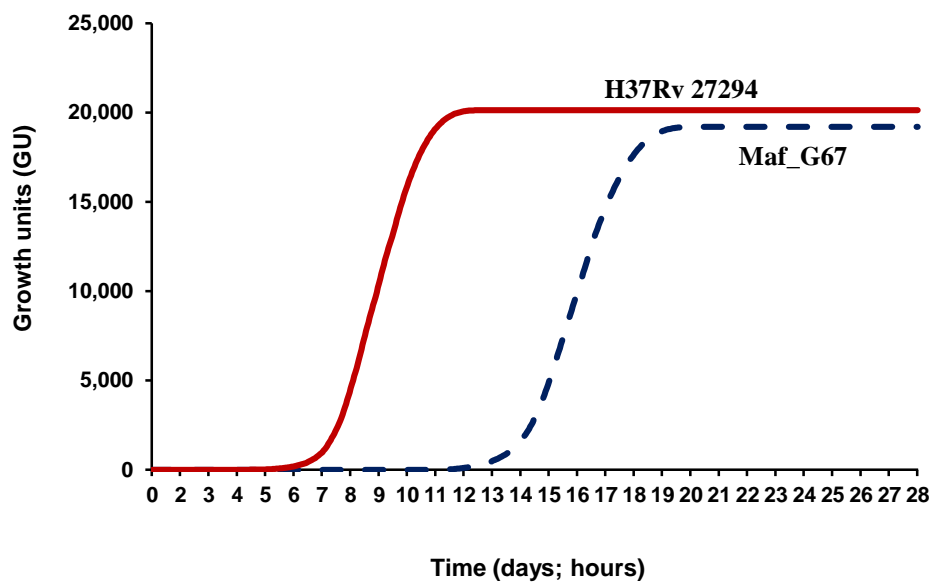

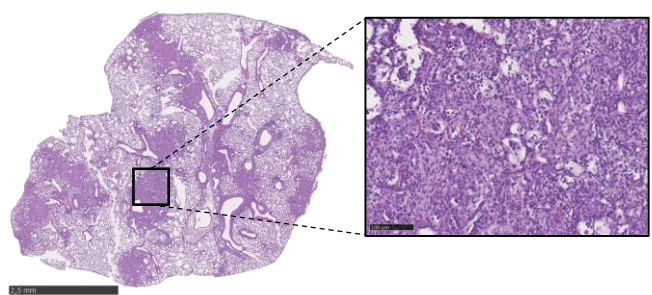

**C57BL/6**  
**HN878 high dose aerosol infection**  
**Day 30 pi**

Supplement: FIGURE S1 — Gating strategy used to delineate the cellular populations present in the lung. [file Data_Sheet_1.PDF]
